# Gender Differences in Axial Spondyloarthritis: Women Are Not So Lucky

**DOI:** 10.1007/s11926-018-0744-2

**Published:** 2018-05-12

**Authors:** T. Rusman, R. F. van Vollenhoven, I. E. van der Horst-Bruinsma

**Affiliations:** Amsterdam Rheumatology and Immunology Center, Amsterdam, The Netherlands

**Keywords:** Axial spondyloarthritis, Gender, Sex, Disease manifestation, TNF inhibitors, Radiological progression

## Abstract

**Purpose of Review:**

Ankylosing spondylitis (AS) was historically seen as a predominantly male disease. However, more recent data showed a more homogenous sex prevalence. Unfortunately, in many studies in axial spondyloarthritis (axSpA), the number of women included is low and the analyses are often not stratified for gender distribution. The purpose of this review is to aggregate the existing data on gender differences in axSpA in order to increase the awareness that female axSpA patients are still under-recognized.

**Recent Findings:**

Several studies considering gender differences revealed that female axSpA patients had different disease manifestations due to different immunological, hormonal, and genetic responses. For instance, allelic frequencies of the AHNK-gene and tissue non-specific alkaline phosphatase (TNAP) haplotypes differed between men and women with ankylosing spondylitis (AS). In addition, different levels of tumor necrosis factor (TNF), interleukins IL-6, IL-17, and IL-18, were found between the two sexes. Furthermore, female patients show a higher diagnostic delay compared to males. Several studies indicate a higher frequency of extra-articular manifestations (EAM) in female axSpA patients, such as enthesitis, psoriasis, and inflammatory bowel disease (IBD), whereas acute anterior uveitis is more prevalent in male patients. Male AS patients more frequently show a higher Bath Ankylosing Spondylitis Radiology Index (BASRI) scores and modified Stoke Ankylosing Spondylitis Spine Scores (mSASSS) than females, which indicates that males have higher radiological damage and radiographic progression. However, disease activity (BASDAI) and quality of life (AsQol) scores are significantly higher in women, and more importantly, they have significantly lower response rates to treatment with TNF inhibitors (TNFi) and a significantly lower drug adherence.

**Summary:**

Despite the fact that men with axial SpA have a worse radiologic prognosis, women have a high disease burden, in part because they have a longer delay in diagnosis, higher disease activity, and significantly less responsiveness to treatment with TNFi.

## Introduction

There is increasing evidence that in drug development, any potential sex and gender differences in general have not been addressed sufficiently, and sometimes never addressed at all [1••, 2]. For example, in vitro experiments are performed in cell lines with unknown XX/XY karyotype, animal experiments are only performed in male mice (in order to prevent “hormonal disturbances”), women are underrepresented in phase 1 and 2 trials (despite significant differences in pharmacodynamics and pharmacokinetics between men and women), phase 3 trials are not powered to detect gender differences in efficacy or toxicity, and post-marketing studies are not always analyzed after stratification for sex [1••, 2]. Recently, sex and gender differences in several diseases are being recognized, such as clinical presentation of cardiovascular disease [3].

First, the terms sex and gender, which are often used interchangeably, need clarification. The term “sex differences” is used to describe differences in biological processes between males and females, such as hormonal, genetic, and immunological functions in a disease. The term gender can be used for describing a person’s self-perception as male or female, and behavioral expression (such as coping style and disease perception) [4]. Thus, besides cardiovascular diseases, gender differences in disease manifestations have also been described in rheumatic diseases, including spondyloarthritis (SpA).

SpA is a chronic inflammatory rheumatic disease that can be divided into predominantly axial and predominantly peripheral forms [5]. Axial SpA (axSpA) encompasses ankylosing spondylitis (AS) with radiological signs of sacroiliitis, and a type without radiographic sacroiliitis, initially called “spondylitic disease without radiographic sacroiliitis” [6] and now renamed “non-radiographic axial SpA” or nr-axSpA [5, 7]. Nr-axSpA might progress to AS within several years [5, 8, 9]. Historically, the male-female prevalence ratio showed a large overestimation favoring males, especially among AS patients; for example, initial studies showed a male-female ratio of 10:1 [10–12]. Subsequently, this ratio has been reported to be approximately 3:1 [13–19]. The most current study reports a steady decline in the male-female ratio among patients with AS/axSpA in Switzerland from 2.57:1 in 1980, down to 1.03:1 by the end of 2016 [20]. In contrast to AS, nr-axSpA patients show hardly any difference in its prevalence among males and females [5, 21–23].

Characteristic symptoms of SpA are inflammatory back pain and progressive functional limitations. In addition, extra-articular manifestations (EAM’s) can develop, such as anterior uveitis (30–40%), psoriasis (10%), and inflammatory bowel disease (IBD, 5–10%) [24]. Since the introduction of TNF alpha inhibitors (TNFi), the treatment of axSpA has improved dramatically [25]. Although the large benefits of this treatment for many axSpA patients, currently, several studies show gender and sex differences in treatment response and adherence and in several other aspects of axSpA, such as disease manifestations and disease burden [23, 26••, 27–33].

Despite accumulating study results considering gender and sex differences in axSpA, female patients are still underrepresented in clinical research [1••]. This under-recognition results in delay in diagnosis, which causes under-diagnosis and delay of optimal treatment strategies, which leads to increased disease burden in female axSpA patients.

The aim of this review is to aggregate the existing data on gender and sex differences in axSpA, in order to increase the awareness of female axial SpA patients, since there is still under-recognition.

## Immunology and Genetics

Immunological and genetic data showed clear sex dimorphisms in response and expression (Table 1). Recent publications revealed a sex difference in immune response of the cytokine TNFα and the interleukin Il-17A. Male axSpA patients showed significant elevated levels of TNFα and Il-17A compared to female patients [34•]. In addition, in AS patients with syndesmophytes, men had significantly higher IL-18 levels, whereas women showed significantly elevated IL-6 [35]. Moreover, sex differences were also found in gene expression in AS patients. One study identified a sex-specific gene expression profile, showing 291 genes uniquely expressed in female AS patients, 1522 genes expressed in males with AS, and 650 genes expressed in both male and female AS patients compared with healthy matched controls [34•]. Another study revealed that different loci of the ANKH gene were associated with AS in male versus female patient [37]. The ANKH gene encodes a progressive ankylosing protein, which is involved in the structural damage in axSpA patients. Moreover, sex differences were found in TNAP (tissue-nonspecific alkaline phosphatase) haplotype, which interplay with the ANKH gene in ossification. This specific TNAP haplotype was associated with AS in men, but not with women with AS [36].

**Table 1:** Sex differences in immunological, hormonal, and genetic aspects in Axial SpA

Author	AS or axSpA	Study design	M/F	Observations
Immunological markers				
Gracey, 2016 [34•]	AS	Observational Cohort	53/41	↑ IL-17A levels + TH17 cells only in male AS patients* ↑ TNF levels in only male AS patients*
Huang, 2012 [35]	AS	Cross-sectional	68/19	↑ IL-18 levels in only male AS patients* ↑ TNF levels only in male AS patients* ↑ IL-6 levels in only female AS patients*
Genetic markers				
Tsui, 2007 [36]	AS	Cross-sectional		TNAP haplotype rs3767155 (G)/rs3738099 (G)/ rs1780329 (T) is a genetic marker associated with AS only in men*
Tsui, 2005 [37]	AS	Cross-sectional		ANKH genetic markers at 5′ end of the gene are associated with AS in affected women; haplotype: rs28006 [C] and rs25957 [C]* ANKH genetic markers at the 3′ end of the gene are associated with AS in affected men; haplotype: rs26307 [C] and rs27356 [C]*
Sex steroids				
Jeong, 2017 [38]	axSpA	Experimental	Female mouse models	↑ estrogen levels suppressed arthritis in female SKG mice (SpA model)*
Mahendira, 2014 [39]	AS	Cross-sectional	0/571	Exogenous estrogens are not associated with initiation or severity of AS in women
Aydin, 2005 [40]	AS	Cross-sectional	58/0	Possible relation between low dehydroepiandrosterone (DHEAS) and bone loss in male AS patients
Giltay, 1998 [41]	AS	Case-control	50/10	Serum testosterone levels are not elevated in male AS patients Other sex steroids differ not between patients and controls
Jimenez-Balderas, 1990 [42]	AS	Case-control	0/17	Exogenous estrogen levels suppressed arthritis and lower clinical disease activity

*M* male; *F* female; *AS* ankylosing spondylitis; *axSpA* axial spondyloarthritis; *SpA* spondyloarthritis; *IL-6* interleukin 6, pro-inflammatory cytokine; *IL-17A* interleukin 17, pro-inflammatory cytokine; *IL-18* interleukin 18, pro-inflammatory cytokine; *TH17 cells* T-helper 17 cells; *TNF* tumor necrosis factor; *TNAP* tissue non-specific alkaline phosphatase; *ANKH gene* progressive ankyloses protein

*Significant gender/sex differences

In addition, sex hormones might also play a role. Estrogen has an anti-inflammatory effect on SpA manifestations by inhibiting TNF alpha production, although contradicting results were presented [2]. One older study revealed a decrease in arthritis and clinical activity in 17 female AS patients after oral estrogen therapy [42]. They also demonstrated that in premenopausal female patients with active AS, the estrogen levels were lower compared to females with inactive disease and significantly lower compared to controls. Also, in postmenopausal AS patients, estrogen levels were lower compared to controls. However, a more recent study showed neither difference in onset nor severity in 571 female AS patients, of which 448 women had used oral estrogen therapy and 123 did not [39]. In a study with a mouse model, the female mice with high estrogen levels had significantly less severe arthritis and SpA manifestations (such as spondylitis, enthesitis, and bowel inflammation) compared to mice with low estrogen levels [38]. In a review and case-control study of 50 males and 10 female AS patients, Giltay et al. [42] described that serum testosterone levels were not elevated in AS patients compared to controls and did not seem to influence progression of AS [41]. However, the precursor of both testosterone and estradiol, dehydroepiandrosterone (DHEAS), which enhances the Th1 immune response, might play a role in the onset and severity of the AS (Table 1). Furthermore, the review revealed that the role of sex steroids in the pathogenesis of AS needs further investigation [41, 43].

These results considering sex differences in immune response, genetic associations and sex hormones, show biological mechanisms, which might contribute to different disease manifestations, disease perception, and treatment response in men and women with axSpA.

## Delay in Diagnosis

The age of onset of AS does not differ between males and females [44, 45], but female seem to have a relatively longer delay in diagnosis (Table 2). For example, this delay in 1976 was reported to be approximately 10 years in female versus 3 years in male patients [13]. Later observations showed a median delay of 9 to 14 years in female and 5 to 7 in male patients [18]. A recent meta-analysis covering a total of 42 studies including 23,889 patients (32.3% women) showed a significantly longer delay in diagnosis among female patients compared to males, 8.8 versus 6.5 years, respectively, with a significant overall effect of all included studies of approximately 0.6 years (*p* < 0.0001; 95%CI 0.31–0.89) [58•]. Only one study revealed a higher diagnostic delay in males compared to females, 9.9 and 6.3 years, respectively [47]. Incidentally, among AS patients as a whole, the median delay in diagnosis is significantly longer in HLA-B27-negative patients (11.4 years) than among those who possess this gene (8.5 years) [45].

**Table 2:** Gender differences in diagnostic delay of axial SpA

Study	AS or axSpA	Study design	M/F	Delay in diagnosis (years) M/F
Mogard, 2017 [46]	axSpA	Cross-sectional	128/55	AS 7.6/8.6 USpA 6.7/6.1
Bandinelli, 2016 [47]	AS	Retrospective	91/44	9.9/6.3*
Webers, 2016 [48•]	AS	Prospective observational cohort	154/62	8.0/10.8*
Landi, 2016 [49]	AS	Observational cohort	817/255	8.9/7.8
Shahlaee, 2015 [50]	AS	Prospective cohort	253/67	8.0/8.8
Bodur, 2012 [51]	AS	Prospective observational cohort	1038/343	4.9/5.3
Yacoub, 2012 [52]	AS	Cross-sectional	87/43	4.6/4.8
Slobodin, 2011 [53]	axSpA	Cross-sectional	79/72	5.9/5.7
Roussou, 2011 [54]	axSpA	Prospective cohort	150/293	5.6/6.3
Atagunduz, 2010 [55]	AS	Cross-sectional	139/96	6.2/7.4
Dincer, 2008 [56]	AS	Cross-sectional	103/8	5.3/14.4
Reed, 2008 [57]	AS	Cross-sectional	91/35	7.3/10.2

Diagnostic delay: first manifestation of the disease symptoms until time of diagnosis

*F* female, *M* male, *AS* ankylosing spondylitis, *axSpA* axial spondyloarthritis, *USpA* undifferentiated SpA

*Significant gender/sex differences

Several reasons have been described to explain the longer diagnostic delay among females, such as the known differences in the presenting clinical symptoms reported by female patients, such as a lower frequency of typical inflammatory back pain as one of the presenting manifestation, more prominent upper thoracic and neck or wide spread pain, along with less severe or slower progression of radiographic damage [53]. Female patients who report wide spread pain are twice as likely to have a delayed diagnosis compared to those without this symptom [53]. The group with wide spread pain was frequently misdiagnosed with fibromyalgia, since it has some overlapping symptoms with axSpA [59]. Almost 25% of female axSpA patients were misdiagnosed at first, although both male and female patients had nonspecific low back pain as pre-SpA diagnosis. This could be the result of physicians’ bias that axSpA is mostly a male disease, and their lack of knowledge of the different disease manifestation in female patients [53].

In conclusion, despite the improvement in delay of diagnosis in women with axSpA, there is still a longer delay and more often misdiagnosis in women, which increase the disease burden in the female patient group.

## Extra-Articular Manifestations

Female gender was found to be positively associated with several extra-articular manifestations (EAM) [60, 61] (Table 3), but others found no differences [50, 53, 68]. Conflicting results have also been reported regarding gender differences in occurrence of acute anterior uveitis (AAU), the most common EAM. It seems to occur more commonly among male patients [48•, 65, 67], but a systematic literature review reported occurrence of AAU to be 28.5% in males versus 33.3% among females [69]. However, this study made no distinction between different types of uveitis, which is important because intermediate and posterior uveitis are unusual in SpA, and more often associated with other diseases, such as sarcoidosis.

**Table 3:** Sex differences in extra-articular manifestations in axial SpA

Study	AS or axSpA	Study design	M/F	Anterior Uveitis M/F	Enthesitis M/F	IBD M/F	Psoriasis M/F
Ibanez, 2017 [62••]	AS	Prospective cohort	25/16	24%/18.8%	NR	4%/0	12%/12.5%
Lubrano, 2017 [63]	axSpA	Retrospective	228/93	NR	MASES 0/1*	NR	NR
Kilic, 2017 [64]	axSpA	Cross-sectional observational cohort	221/139	34%/19%	NR	3%/2%	16%/7%
Webers, 2016 [48•]	AS	Prospective observational cohort	154/62	18.2%/18.0%	MEI 13.5%/18.7%*	7.8%/4.9%	3.9%/4.9%
Landi, 2016 [49]	axSpA	Observational cohort	817/255	23.9%/23.4%	41.1%/67.9%*	NR	NR
Shahlaee, 2015 [50]	AS	Prospective cohort	253/67	15.8%/13.4%	68.8%/82.1%*	7.5%/7.5%	4.7%/3.0%
Zarco, 2015 [60]	AS	Prospective observational cohort	379/222	14.0%/13.1%	NR	5.0%/5.4%	24.8%/32.9%*
Mitulescu, 2015 [65]	axSpA	Retrospective	81/45	12.3%/2.2%*	NR	NR	NR
Tournadre, 2013 [61]	axSpA	Prospective cohort	239/236	NR	MASES 1.4/3.4*	NR	NR
Carvalho, 2012 [66]	AS	Observational cohort	1090/415	19.8%/16.8%	Enthesitis 28%/25% MASES 2.0/2.42*	NR	13.8%/29.1%*
Yacoub, 2011 [52]	AS	Cross-sectional	87/43	NR	MEI 5.2/7.7*	NR	NR
Attagunduz, 2010 [55]	AS	Cross-sectional	139/96	20.6%/26.2%	36.4%/64.8%*	NR	NR
Braakenburg, 2008 [67]	HLA-B27 associated AAU	Retrospective	96/81	54%/46%	NR	NR	NR

*M* male, *F* female, *NR* not reported, *MASES* Maastricht Ankylosing Spondylitis Enthesitis Score, *MEI* Mander Enthesitis Index, *IBD* inflammatory bowel disease, *axSpA* axial spondyloarthritis, *AS* ankylosing spondylitis, *HLA-B27* human leukocyte antigen B-27, *AAU* acute anterior uveitis

*Significant gender differences

Enthesitis is more common and more severe among in female patients [49, 50, 52, 61, 63, 66]. This finding could be an explanation for the same or even higher disease burden in female patients compared to males, despite their slower radiological progression. Three studies, including a systematic review and meta-analysis, indicate that female patients experience more inflammatory bowel disease (IBD) compared to male patients [60, 61, 70]. In addition, there are some studies that showed a higher prevalence of psoriasis in female axSpA patients compared to males [60, 65].

Overall, although there are some conflicting results, it seems that female patients more frequently have enthesitis and IBD, whereas male patients may have AAU more frequently.

## Disease Activity and Severity

### Disease Activity Scores

Female axSpA patients showed a higher disease burden concerning disease activity and pain scores (Table 4). Reported were significantly higher Bath Ankylosing Spondylitis Disease Activity Index (BASDAI) scores in females compared to males, of which the items fatigue, total back pain, and longer duration of morning stiffness showed the largest differences [26••, 27, 30, 48•, 49, 50, 52, 54, 61, 66]. Only one study showed higher Bath Ankylosing Spondylitis Functionality (BASFI) scores in female patients, whereas the other studies showed no gender difference [61]. The limited data available considering the AS Disease Activity Score (ASDAS) showed no gender differences [26••, 48•]. Studies on sex differences in CRP levels showed significantly higher baseline levels in male patients compared to females [26••, 48•, 50, 61, 63]. Data on ESR levels were inconclusive to identify sex differences.

**Table 4:** Gender differences in disease activity scores, functionality scores, severity, and extra spinal involvement in axial SpA at baseline

Study	AS or axSpA	M/F^◊^	Study design	Disease duration (years)^◊^	Age (years)^◊^	TNF naive^◊^	BASDAI^◊^	ASDAS-CRP^◊^	BASFI^◊^	QoL^◊^	CRP level^◊^	ESR level^◊^
Ibanez, 2017 [62••]	AS	25/16	Prospective cohort	5/3	43.1/41.7	25/16	5.1/ 5.2	NR	NR	NR	6/4.5	NR
Lubrano, 2017 [63]	axSpA	228/93	Retrospective	NR	NR	NR	5.7/6.1	3.7/3.4*	5.5/5.5	NR	1.3/1*	NR
Kilic, 2017 [64]	axSpA	221/139	Cross-sectional observational cohort	NR	36/37.4	n.a.	3.3/4.2*	2.6/2.7	2.5/2.8	7.1/8.9*	16.1/12.5	18.9/24.9*
Landi, 2016 [49]	AS	817/1072	Observational cohort	16/15.9	40.9/43.3*	n.a.	4.1/4.8*	NR	4.6/4.8	6.9/8.3*	NR	NR
Vargas, 2016 [71•]	axSpA	81/87	Observational cohort	NR	29.9/30.5	NR	3.6/4.3*	2.3/2.5	NR	NR	3/3	NR
Shahlaee, 2015 [50]	AS	253/67	Prospective cohort	15.5/15.6	37.6/39.5	n.a.	4.6/5.0	NR	3.8/4.3	7.7/8.5	18.7/10.6*	17.6/18.6
Webers, 2015 [48•]	AS	154/62	Prospective observational cohort	n.a.	42.3/46.8	n.a.	3.2/3.9*	2.7/2.8	3.5/3.2	5.8/7.2	19.5/14.2*	14.5/14.8
Gremese, 2014 [23]	axSpA	118/52	Retrospective	16.5/16.1	39.2/40.3	118/52	5.5/5.6	NR	NR	NR	NR	NR
Tournadre, 2013 [61]	axSpA	239/236	Prospective cohort	n.a.	31.9/34	239/236	4.0/4.6*	2.9/3.0	2.7/3.3*	8.0/10.2*	11/6.9*	NR
Horst-Bruinsma, 2012 [26••]	AS	957/326	Pooled data clinical controlled trials	9.4/7.4*	31.2/35*	642/225	58.6/62.7*	3.7/3.6	55.8/57.5	62.4/66.2*	20.9/13.1*	NR
Carvalho, 2012 [66]	axSpA	1090/415	Observational cohort	13.9/30.3*	41/45*	n.a.	4.0/4.6*	NR	4.5/4.8	7.5/8.3*	NR	NR
Yacoub, 2012 [52]	AS	87/43	Cross-sectional	9.5/9.1	27.9/28.8	n.a.	43.1/48.8*	NR	53/54.2	NR	28.5/35.2	44.3/43.7
Roussou, 2011 [54]	axSpA	172/344	Prospective cohort	9.7/10.1	46.5/47.6	n.a.	5.7/6.3	NR	4.9/5.2	NR	7.6/8.3	14.3/20.3*
Cansu, 2011 [68]	AS	66/36	Prospective cohort	n.a.	n.a.	n.a.	NR	NR	NR	NR	M = F^a^	NR
Bodur,2010 [51]	AS	1038/343	Prospective observational cohort	n.a.	n.a.	n.a.	3.7/4.2*	NR	3.3/3.2	6.8/7.3	NR	NR
Jung, 2010 [72]	AS	434/71	Registry	9.9/7.7*	29.8/31.5	n.a.	M = F^a^	NR	NR	NR	NR	NR
Lee, 2007 [73]	AS	302/100	Cross-sectional	32/31.5	55.5/53.0	n.a.	NR	NR	43.3/49.0*	Worse F*^a^	NR	NR

*F* female, *M* male, *QoL* quality of life, *BASDAI* Bath Ankylosing Spondylitis Disease Activity Index, *ASDAS* Ankylosing Spondylitis Disease Activity Score, *BASFI* Bath Ankylosing Spondylitis Functionality Index, *CRP* C-reactive protein, *ESR* Erythrocyte Sedimentation Rate, *NR* not reported, *TNF naive* no TNF use before start of the study data collection, *n.a.* no TNF inhibitor treatment as study medication

*Significant gender/sex difference

^◊^Only descriptive data present; male/female

### Quality of Life

Female gender corresponded with a significantly lower quality of life (QoL) according to the ASQoL (questionnaire for QoL in AS) compared to male patients in most studies [48•, 49, 50, 66] (Table 4). However, other QoL questionnaires, such as EuroQL and SF-36 scores, showed no (large) differences over time [48•]. Female axSpA patients also showed higher Bath Ankylosing Spondylitis Global scores (BASG) [54, 61, 66], which indicate that female patients experienced worse overall well-being in daily life compared to males. These results are consistent with observations done in rheumatoid arthritis and other autoimmune diseases, where female patients have worse QoL scores compared with males despite equal control of inflammation [74].

### Differences in Radiological Progression

Overall, most studies were small but showed that men had worse radiological progression compared to women, although one study showed the opposite (Table 5) [61]. Frequently, male sex was indicated as a prognostic factor for worse and more severe radiological progression including development of syndesmophytes, measured with the BASRI-spine and mSASSS scores [73, 77, 80–82, 85]. Additionally, hip involvement seems to be worse in male patients [68, 72]. However, more extensively research revealed a small nuance in radiological progression. Female patients seem to have higher progression in the cervical spine and males in the lumbar spine, thereby female patients showed slow radiological progression and males fast progression [76]. This slower radiological progression in women probably results in more nr-axSpA diagnoses in female axSpA patients compared to males [86]. Despite the lower radiological progression, the disease burden in female patients is still high. Several studies confirmed that nr-axSpA patients (both male and female) showed the same levels of disease activity, pain, and impaired function as “radiological axSpA” or AS patients [87].

**Table 5:** Sex differences in radiological progression in Axial SpA

Study	AS or axSpA	Study design	M/F	Disease duration M/F	Age M/F	Time measurement radiological progression (months)	Radiological progression/damage M/F
1A. Radiological progression and damage according to BASRI							
Landi, 2017 [49]	AS	Observational cohort	817/1072	16/15.9	40.9/43.3	At baseline	7.3/5.8*^a^
Carvalho, 2012 [66]	axSpA	Observational cohort	172/344	13.9/30.3	41/45	n.a.	7.6/5.5*^a^
Yacoub, 2011 [52]	AS	Cross-sectional	87/43	9.5/9.1	n.a.	One time point	8.1/6*^b^
Attagunduz, 2010 [55]	AS	Cross-sectional	139/96	12.1/12.1	37.2/41.8	One time point	1.77/0.97*^b^
Lee, 2007 [73]	AS	Cross-sectional	302/100	32/31.5	55.5/53.0	One time point	10/6.5*^b^
1B. Radiological progression and damage according to mSASSS							
Webers, 2015 [48•]	AS	Prospective observational cohort	154/62	n.a.	42.3/46.8	At baseline	13.8/6.5*
Tournadré, 2013 [61]	axSpA	Prospective cohort	239/236	1.5/1.6	31.9/34	At baseline	1.45/2.9
1C. Radiological progression and damage according to other scoring methods							
Tournadré, 2013 [61]	axSpA	Prospective cohort	239/236	1.5/1.6	31.9/34	At baseline	More male patients had sacroiliitis compared to females: 45%/33%
1D. Radiological progression and damage presented only as descriptive data							
Maas, 2015 [75]	AS	Prospective longitudinal observational cohort	121/55	n.a.	n.a.	At baseline and 24, 26 and 48 months	Increased radiological progression (mSASSS) in male patients
Baraliakos, 2011 [76]	AS	Retrospective	114/32	n.a.	n.a.	24 months	Male patients experienced increased overall radiological progression. Female patients experienced increased progression in the cervical spine
Tubergen, 2011 [77]	AS	Retrospective	94/48	n.a.	n.a.	48 months	Increased radiological progression (mSASSS) in male patients
Cansu, 2011 [68]	AS	Prospective cohort	66/36	n.a.	n.a.	One time point	Increased radiological progression (BASRI-t) in male patients
Jang et al. 2011 [78]	AS	Prospective study	556/213	n.a.	48/45	10 years	Male patients had more severe sacroiliitis compared to females
Jung, 2010 [72]	AS	Registry	434/71	9.9/7.7*	34.9/35.4	One time point	More male patients developed bamboo spine
Aggarwal, 2009 [79]	AS	Cross-sectional	n.a.	n.a.	n.a.	One time point	No radiological differences between male and females
Rudwaleit, 2009 [80]	AS	Cross-sectional	151/85	n.a.	n.a.	At one time point	Male patients had a significant higher mSASSS compared to female patients
Ward, 2009 [81]	AS	Prospective	298/100	n.a	n.a	One time point	Increased radiological progression (BASRI-s) in male patients
Boonen, 2009 [82]	AS	Review	n.a.	n.a.	n.a.	n.a.	Male patients presented themselves with more ankylosis and syndesmophytes compared to females
Calin, 1999 [83]	AS	Cross-sectional	351/72	n.a.	n.a.	24 months	Male patients show more radiological progression compared to females
Gran, 1984	AS	Retrospective	60/22	13.7/15.3	40.6/36.7	One time point	Males showed significantly more often radiological involvement of the lumbar spine
Kidd, 1988 [84]	AS	Cross-sectional	70/35	17.7/16.2	n.a./42.8	One time point	Male had significantly greater spinal radiological changes compared to females Male and females had equal sclerosis, erosions and ankylosis
Spencer, 1979 [51]	AS	Cross-sectional	164/36	n.a.	n.a.	One time point	No radiological differences between males and females

*M* male, *F* female, *BASRI* Bath Ankylosing Spondylitis Radiology Index, *BASMI* Bath Ankylosing Spondylitis Metrology Index, *mSASSS* modified Stoke Ankylosing Spondylitis Spine Score

*Significant sex difference

^a^BASRI-total score

^b^BASRI-spine score

◊BASRI-SI score

## Treatment Response and Drug Adherence

Overall, treatment efficacy of TNFi is significantly lower in women compared to men with axSpA, and they have a significantly lower drug adherence (Table 6). In most randomized controlled trials, these gender differences in efficacy were not shown due to the relatively low numbers of women included and because most studies were only powered for efficacy of the drug and not to detect gender differences in response. However, if the data of these trials were reanalyzed after stratification for gender, a significantly lower level of response (ASDAS and BASDAI) and treatment adherence in females was found [23, 26••, 27, 28, 30–33, 63, 91] (Table 6). These studies indicate that women with AS have doubled risk at lower drug adherence of TNFi compared with males. In addition, significantly more female patients switched TNFi treatment, which indicate worse treatment adherence in female patients [90, 92]. This may also imply a weaker treatment response.

**Table 6:** Gender differences in TNFi treatment response and adherence

Study	AS or axSpA	Study design	M/F	Treatment response M/F	TNF naive population	Follow-up period
Lubrano, 2017 [63]	axSpA	Retrospective	228/93	ASAS40%: ↑ response male*‡	Yes	Every 3 months
Rusman, 2017 [88]	AS	Prospective observational cohort	194/97	BASDAI50%: 62%/43%* BASDAI50%: 59%/46%* ASDAS: 64.9%/47.6%* ASDAS: 65.9%/46.3%*	Yes	12 months 24 months 6 months 12 months
Lorenzin, 2015 [31]	AS	Retrospective	52/18	ASAS20: 82.9%/65.7%	n.a.	60 months
Gremese, 2013 [23]	axSpA	Retrospective	118/52	BASDAI50%: 67.8%/46.2%*	Yes	12 months
Horst-Bruinsma, 2012 [26••]	AS	Pooled data clinical controlled trials	957/326	ASDAS: 89.4%/68.4%*	Yes	12 weeks
Paccou, 2012 [32]	AS	Retrospective	121/68	BASDAI50%: 78.5%/21.5%*	Yes	3 months
Arends, 2011 [28]	AS	Prospective longitudinal observational	152/68	ASAS20: ↑ response Male*‡ ASAS40: ↑ response Male*‡	Yes	3 and 6 months 6 months
Glintborg, 2010 [27]	AS	Observational cohort	364/239	Change in BASDAI: 27/22	Yes	6 months
Study	AS or axSpA	Study design	M/F	Treatment adherence	Study time period	
Rusman, 2016 [89]	AS	Prospective cohort	74/48	Males: 44.9 months Females: 33.4 months	Mean 4.8 years	
Horst-Bruinsma, 2012 [26••]	AS	Pooled data clinical controlled trials	957/326	↓ Females: HR: 1.5	12 weeks	
Glintborg, 2013 [90]	AS	Observational cohort	1076/360	↑ Males: HR:1.76	10 years	
Arends, 2011 [28]	AS	Prospective longitudinal observational	152/68	↓ Females: HR:0.41	6 months	
Glintborg, 2010 [27]	AS	Observational cohort	364/239	↓Females: HR:3.4	5 years	
Kristensen, 2010 [30]	AS	Prospective observational cohort	182/61	↑Males: HR:0.36	2 years	
Pavelka, 2009 [33]	AS	Prospective observational	238/72	↓Females: RR: 2.2	2 years	

Treatment adherence = time on TNFi

*AS* ankylosing spondylitis, *axSpA* axial spondyloarthritis, *ASAS20* ASAS response criteria, *ASDAS* Ankylosing Spondylitis Disease Activity Score, *BASDAI* Bath Ankylosing Spondylitis Disease Activity Score, *BASDAI50%* response op de BASDAI 50%, *TNF naive* no earlier use of TNFi treatment before study, *HR* hazard ratio

*Significant gender difference

Some predictors were associated with a better treatment response, such as the presence of the HLA-B27, absence of enthesitis, short disease duration, and being TNFi naive [33, 92]. Interestingly, these predictors were negatively associated with female gender, because women with AS have a higher prevalence of enthesitis and had a longer delay in diagnosis (Tables 2 and 3). These factors may contribute to the gender differences in TNFi adherence and response.

Currently, gender differences in treatment response are still a dilemma. However, almost no specific studies considering gender and sex differences were performed. A re-analysis of randomized clinical trials, which are powered for efficacy and toxicity between the studied drug (or placebo), and not for sex differences, has only been done in three TNFi studies with etanercept, adalimumab, and infliximab, but not for all the other TNFi nor for the interleukin 17 blocker secukinumab [88, 89].

Recently, several hypotheses for gender and sex differences in response to TNFi were formulated, such as difference in body composition. Females have, in general, higher fat percentages compared to males, and different gonadal hormones and even different gene expressions compared to male axSpA patients [1••].

Several studies showed that a higher body mass index (BMI) resulted in a lower TNFi treatment response [23, 71•], of which one of the studies even found a correlation between BMI and the inflammation marker CRP in female axSpA patients [71•]. In addition, other data revealed that there was a significant relationship between female AS patients with high disease activity scores (ASDAS and BASDAI) and a high body fat percentage (BF%) or fat mass index (FMI), as contradicting male patients with high disease activity scores had low BF% and FMI [29]. Overall, women in general have a higher BF% compared to males, which might be an explanation for the worse TNFi treatment response in female axSpA patients [62••]. As mentioned previously, male axSpA patients showed a significantly elevated level of TNFα compared to female patients, which might be another possible explanation for a worse TNFi treatment response in female axSpA patients.

## Conclusion

Despite the fact that male axSpA patients has more radiographic damage compared to females, female patients have a higher disease burden due to a longer diagnostic delay, higher disease activity, and a lower efficacy of treatment. Although there is increased recognition, of sex and gender differences in axSpA, this review also demonstrated a persistent lack of comprehensive knowledge about disease manifestations in female patients. Further studies into sex and gender differences in the manifestation of axSpA may result in less under-diagnosis and misdiagnosis, more optimal treatment strategies, and decreased overall disease burden in female axSpA patients.
